# Physical and chemical properties, structural characterization and nutritional analysis of kefir yoghurt

**DOI:** 10.3389/fmicb.2022.1107092

**Published:** 2023-01-11

**Authors:** Ran Xiao, Ming Liu, Qing Tian, Ming Hui, Xin Shi, Xiaoge Hou

**Affiliations:** ^1^College of Biological Engineering, Henan University of Technology, Zhengzhou, Henan, China; ^2^Industrial Microorganism Preservation and Breeding Henan Engineering Laboratory, Zhengzhou, Henan, China

**Keywords:** kefir, SEM, CLSM, flavour, amino acid

## Abstract

Scanning electron microscopy (SEM), Confocal laser scanning microscopy (CLSM) and low field nuclear magnetic resonance (LF-NMR) were used to analyse the relationship between the chemical, texture, rheology, microstructure and water distribution of kefir (yeast, acetic acid bacteria and *Lactobacillus plantarum*) yoghurt fermented by mixed bacteria and *L. plantarum* L_1_ fermented yoghurt. This work was conducted to prepare a real champagne yoghurt and explore the difference between it and ordinary yoghurt. The nutritional evaluation of the two treatment groups was carried out by amino acid analysis, and the volatile flavour substances of the two treatment groups were detected by solid phase microextraction (SPME)–gas chromatograph (GC)–mass spectrometry (MS). Results showed that the addition of acetic acid bacteria and yeast increased the water content of kefir, resulting in a decrease in its water-holding rate. Moreover, the increase in acidity weakened the connection between the protein networks, the flocculent protein structure was not more densely stacked than the L_1_ group, and the internal bonds were unstable. The rheological results showed that the apparent viscosity decreased faster with the increase in shear force. The CLSM and LF-NMR showed that the hydration and degree of freedom of kefir yoghurt protein decreased, resulting in an increased protein network density. The SEM showed that the cross-linking between kefir casein clusters was considerably tight to form small chains, the pore distribution was uneven, and a weak cheese structure was formed. In addition, the volatile flavour substances in the kefir group increased the phenylethyl alcohol, isobutanol, and isoamyl alcohol compared with those in the L_1_ group, with a slight refreshing taste brought by alcohol and special soft malt alcohol aroma and rose aroma not found in ordinary yoghurt, which was more in line with the characteristics and taste of traditional kefir champagne yoghurt.

**Graphical Abstract:**
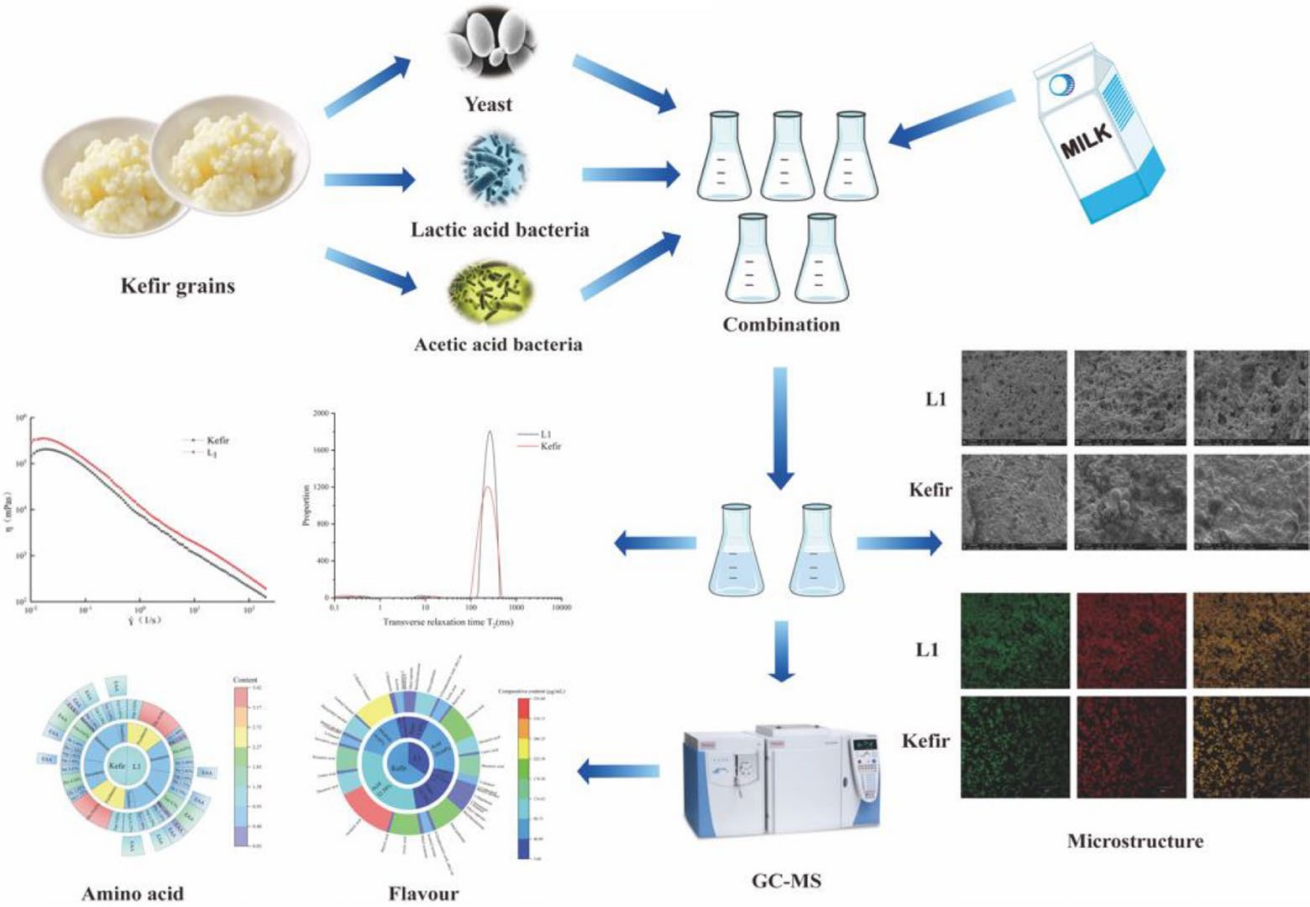

## 1. Introduction

Kefir originated in the Caucasus and is made from cow’s and goat’s milk that has been naturally fermented in sheepskin bags (Petrova et al., 2021). Kefir is widely consumed and popular in many countries, including Europe, Asia, South America and North America (Sadiye, 2020). Moreover, kefir has been consumed for thousands of years because of its health benefits and holds a significant role in food. Kefir is different from yoghurt and other types of fermented dairy products. Yoghurt is fermented from milk and bacteria (yogurt cultures), while kefir is an acidic, low-alcohol probiotic drink derived from a complex mixture of metabolites of bacteria (including *Lactobacillus*, *Lactococcu*s and *Acetate*) and yeast (Lynch et al., 2021; Baniasadi et al., 2022). In addition, kefir differs from other fermented dairy products, which is known as ‘champagne yoghurt’. Given that kefir contains complex symbiotic relationships (lactic acid and acetic acid-producing bacteria, lactose fermentation and alcoholic yeast), such a complex microecological environment allows the lactose, protein and fat in milk to be degraded into galactose, lactic acid, exopolysaccharides, vitamins, free amino acids, free fatty acids, volatile alcohols, aldehydes, ketones and esters, which compounds create kefir’s unique flavour (Souza and Dias, 2017; Sharma et al., 2021). A number of studies have reported the beneficial properties of kefir, such as lowering blood pressure (Silva-Cutini et al., 2019), anti-cancer (Sharifi et al., 2017), anti-viral (Hamida et al., 2021), cholesterol-lowering (Lim et al., 2017), anti-diabetic (Salari et al., 2021), anti-inflammatory (Chen et al., 2020) and immunity-boosting and anti-oxidant (Chen et al., 2020; Patil et al., 2021).

The most abundant flora in kefir granules is lactic acid bacteria, which provides a certain acidity and viscosity of yoghurt. Many lactic acid bacteria are probiotics and have a health-promoting effect (Gezginç et al., 2022). Yeast is the main feature that distinguishes kefir yoghurt from other types of yoghurt. The addition of yeast adds ethanol and carbon dioxide to kefir yoghurt and increases the flavour of yoghurt (Şahingil, 2019). However, an excessive amount yeast can give the yoghurt a yeasty taste and affect the flavour of the product (Farag et al., 2020). Meanwhile, a certain amount of acetic acid bacteria can provide acetic acid for kefir and combine with alcohols to form esters, which also has a certain positive effect on product flavour (Lynch et al., 2019). Natural fermenters are more appropriate for fermenting kefir because the strain of kefir grain is easy to change in the fermentation process (Wang et al., 2016). Currently, kefir grain fermented yoghurt is difficult to replicate and cannot be standardised for production (Nielsen et al., 2014). Accordingly, many researchers have isolated these three types of typical dominant strains for process compounding to find the appropriate amount of co-fermented milk to produce kefir to standardise the production of kefir yoghurt. These researchers have chosen the right method to enrich the volatile components, determine the flavour substances and organoleptically evaluate them to ensure that the right ratio of strains can be determined for subsequent experiments (Xing et al., 2017; Duran et al., 2022). However, few articles on structural characterisation in kefir yoghurt have been published. The three pillars of *Lactobacillus plantarum* L_1_, yeast TN_1_ and acetic acid bacteria A_3_ were selected for the fermentation of kefir yoghurt to find the reason for the change in the structure of kefir yoghurt after the addition of different acetic acid bacteria and yeasts. The physical and chemical indexes, microstructural observation, rheology and texture of kefir yoghurt and single lactic acid bacteria fermented yoghurt were determined, and the nutritional flavour was analysed. This study aims to explore the changes in the structure of kefir yoghurt after the addition of yeast and acetate and to discover the pattern of the unique flavour and structure of kefir yoghurt, which will provide a theoretical basis for the development and quality control of kefir products.

## 2. Materials and methods

### 2.1. Materials

*Saccharomyces cerevisiae* (TN_1_), *L. plantarum* (L_1_) and *Acetobacter tropicalis* (A_3_) were provided by our laboratory. Meanwhile, the milk was purchased from Mengniu Co., Ltd.

### 2.2. Yoghurt fermentation

(1) Strain activation: The A_3_ and L_1_ strains were activated with MRS liquid medium. TN_1_ strain was activated by using YPD liquid medium. (2) Fermentation experiments: Approximately 50 ml of pure milk and 2.5 g of white granulated sugar were added to several 100 ml yoghurt bottles. After pasteurisation (62°C, 30 min), the strains were cooled, inoculated with 7% (*V/V*) of the fermentation agent, mixed and cultured at 37°C for 12 h until the fermentation was completed. Thereafter, the strains were transferred to the 4°C refrigerator and cooked overnight.

### 2.3. pH and titratable acidity (TA)

The pH of the yoghurt was measured with an analyser (Qiu et al., 2021), whilst that of kefir and L_1_ fermented milk was measured with a digital pH meter (*FE28*-Standard, METTLER TOLEDO, America). The titration method adapted from Chouchouli et al. (2013) was applied to determine the TA of the yoghurt samples. Briefly, 10 g of yoghurt was mixed with 20 ml of distilled water and titrated with NaOH (0.1 mol/l) in the presence of phenolphthalein. The results were expressed as a percentage of lactic acid. The determination was carried out in triplicate.

### 2.4. Water-holding capacity (WHC) and syneresis

WHC was determined by using the method previously reported (Nguyen et al., 2017) and slightly modified. he dehydration shrinkage in yoghurt samples was measured with an Eppendorf centrifuge (5810R, Eppendorf, Germany) at 3800 g at 4°C for 30 min for 20 g of yoghurt. Specifically, a 15 g yoghurt sample was centrifuged at 12,000 g at 4°C for 20 min. After centrifugation, the clear supernatant was poured out, weighed and used to determine the percentage of dehydration shrinkage (W/W). All measurements were repeated three times.


(1) $$\text{WHC}\left(\%\right)=\frac{\text{weight of drained gels}\left(g\right)}{\text{weight of yoghurt}\left(g\right)}\times 100$$

(2) $$\text{Syneresis}\left(\%\right)=\frac{\text{weight of whey}\left(g\right)}{\text{weight of yoghurt}\left(g\right)}\times 100$$

### 2.5. Textural analysis

The kefir and L_1_ samples were taken out after ripening overnight in a 4°C refrigerator for comparative analysis. A TA-XT plus texture analyser (Stable Micro System Co., Britain) was used to determine the texture parameters of the samples. During the TPA mode test, P/50 probe is used, the test distance is 25 mm, the trigger point is 5.0 g, the pre-test speed is 2 mm/s, the test speed is 2 mm/s, and the post-test speed is 6 mm/s. The indicators include: hardness, elasticity, cohesion, stickiness and resilience.

### 2.6. Rheological analysis

Before the test, the yoghurt sample was placed at room temperature for 15 min, and the apparent viscosity was measured by using haake rheometer (MARS 60, Thermo Fisher Scientific, American). The selected rotor model was P60/Ti-02150138. Fixture for the test plate (diameter 25 mm) test spacing of 500 μm, 1 ml of yoghurt sample was tested on the surface, and the excess samples were removed. Calibration was performed at 25°C for 1 min, with 30 points at 0.1–200 s^−1^ shear rate (γ) for testing to create a dynamic viscosity curve.

### 2.7. Confocal laser scanning microscopic (CLSM) analysis

A CLSM (FV3000, OLYMPUS, Japan) was used to study the microstructure of yoghurt. Approximately 3 ml of fermented milk was taken, and 30 μl of 0.125% Nile red dye solution (the excitation and emission wavelengths were 580–630 nm) was used to stain the lipid. After colour development, 10 μl of 0.1% *Fast Green* solution (the excitation and emission wavelengths were 641–741 nm) was used to stain the protein for 20 min (Laiho et al., 2017; Wang et al., 2019). Furthermore, 150 μl slides containing the dye mixture were placed under CLSM for observation. Nile Red was used to excite and observe the fat in the yoghurt at 561 nm. *Fast Green* was used to excite and observe the protein in the yoghurt at 640 nm.

### 2.8. Determination of microstructure scanning electron microscopy (SEM)

The microstructure of the yoghurt samples was observed by SEM (Quanta 250FEG, FEI, America). The sample preparation for SEM is performed according to the method described (Bensmira and Jiang, 2012; Pan et al., 2019). The two groups of fermented milk were cut from the middle of the sample square curd and placed in 2% glutaraldehyde solution at 4°C for more than 12 h. After fixation, the samples were washed three times with PBS (pH 7.2) for 5 min each time. Moreover, 50, 70, 80 and 90% gradient ethanol were dehydrated for 10 min after cleaning and dehydrated twice with 100% ethanol for 10 min each. After dehydration with tert-butanol replacement 2 times, each 15 min. After the operation is completed, put in-80°C refrigerator frozen overnight, using vacuum freeze-drying samples, with a blade to cut off the dry part, the debris installed in the aluminum SEM short rod, and sputtering cup pad coated with vacuum gold. Following the examination of the sample’s microstructure by vacuum SEM, a gold film was coated on the surface of the sample with platinum. The final voltage was 15 kV, with magnifications of 5,000 and 20,000 times observed by SEM.

### 2.9. LF-NMR analysis

The L_1_ single bacteria fermented yoghurt and kefir fermented milk were placed into the special sample tube of nuclear magnetic resonance and detected in the nuclear magnetic resonance sample pool. The main parameters of the instrument are set as follows: Micro MR-CL-I low-field NMR analyser (Micro MR-CL-I, Niumag Electronic Technology Co., Ltd., Shanghai, China), 1–10 mm magnet probe and 20 MHz proton resonance frequency. The Carr–Purcell–Meiboom–Gill pulse sequence was applied to collect the T2 relaxation time. The other major parameters were set as follows: the waiting time (WT) was 7,000 ms, the time to echo (TE) was 0.200 ms, the number of echoes (NECH) was 9,000, and the number of scans (NS) was 16. The signal attenuation curve of the transverse relaxation time was obtained.

### 2.10. Determination of free amino acid content

The single free amino acid content (mg/g) was determined with an amino acid analyser (S433D, Sykam, Germany). Chromatographic column: LCA106//Na detection wavelength: 570 mm + 440 mm mobile phase: sodium citrate A = 0.12 N, pH 3.45; b = 0.2 N, pH 10.85. Temperature: 58°C–74°C. Gradient temperature control flow rate: elution pump 0.45 ml/min + derivative pump 0.25 ml/min. Pressure: 30–60 bar (Yang et al., 2021).

### 2.11. GC–MS analysis

The treatment group at the end of fermentation was taken to detect flavour components. Headspace extraction was performed through solid phase microextraction (SPME). Each sample (3 g) of 20 μl of 0.009 g/l 2-octanol was placed in a 20 ml vial as an internal standard for subsequent quantitative analysis. Determination was carried out by using SPME combined with GC–MS. After exposure of the SPME fibres to the top space at 45°C for 45 min for sampling, the SPME fibres were introduced into a GC syringe and allowed to stand for analysis for 3 min for the thermal desorption of the analytes (Shi et al., 2022).

GC–MS was performed with a Thermo Scientific Trace 1,300 gas chromatograph connected to a Thermo Scientific ISO7000 single quadrupole mass spectrometer selective detector (Trace1300*-*I*SQ7000*, Thermo Fisher Scientific, Waltham, MA, United States). A DB-WAX chromatographic column was used (30 m × 0.25 mm inner diameter and 0.25 μm film thickness, J&W Scientific, United States). An ultra-high purity helium was used as the carrier gas at a constant flow rate of 1 ml/min. The separation ratio was 20:1. The column temperature was raised from room temperature to 40°C for 1 min. The temperature was raised to 150°C at 5°C/min and to 210°C at 10°C/min for 5 min. The MS conditions were as follows: 280°C ion source temperature, 215°C transmission line temperature, 70 eV ionisation mode EI and 35–450 u mass-charge (m/z) scan range (Ding et al., 2015). The compounds were identified through MS library searches (NIST/EPA/NIH version 2.0 (1995) and MS data in the Wiley registry) and the MS spectral database library of the National Institute of Standards and Technology.

### 2.12. Statistical analysis

All the experiments were carried out three times. Origin 2018 software was used for data mapping and statistical analysis. IBM SPSS Statistics 26 software was utilised for significance analysis. The significance level of 5% was used and data were shown as mean ± standard error of the mean.

## 3. Results and discussion

### 3.1. Strains observed under an SEM

**Figure 1:**
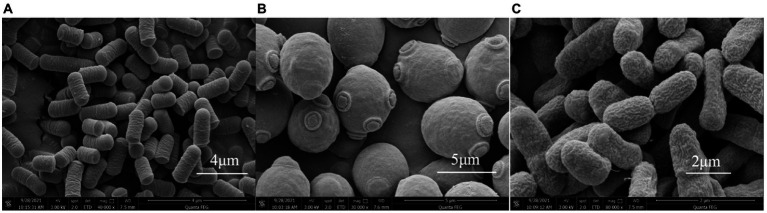
**(A)** L_1_ morphology under the scanning electron microscope. **(B)** TN_1_ morphology under the scanning electron microscope. **(C)** A_3_ morphology under the scanning electron microscope.

The initial strain was observed under an electron microscope. The three strains used to ferment yoghurt are shown in Figure 1. A is *L. plantarum* L_1_, B is *S. cerevisiae* TN_1_, and C is *A. tropicalis* A_3_.

### 3.2. Chemical properties

At the end of fermentation, the pH value and TA of the kefir group were higher than those of the L_1_ group (Table 1). This condition is due to the co-fermentation of the lactic and acetic acid bacteria, resulting in the excessive production of lactic and acetic acid. The water holding rate of the L_1_ group was the highest, and the whey separating rate was lower than that of the kefir group. The decrease in the kefir group may be due to the destruction of the casein network structure by the ethanol produced by the fermented lactose in the yeast group, resulting in a decrease in the stability of the fermented milk gel and the water holding rate. The whey precipitation rate is a bad characteristic of yoghurt. The whey precipitation rate of kefir group is larger, which may be due to the rich variety of organic acids in mixed fermentation. The high pH and titration acidity indicate that the acidity is considerably high. The isoelectric point of casein is easier to approach, which results in coagulation and excessive whey precipitation. As the results of previous studies have shown. Khan et al. (2014) reports that higher acid concentrations cause milk protein denaturation, which significantly affects the binding between them, resulting in milk protein loss as fine particles.

**Table 1:** Comparison of physicochemical indexes of different yoghurt groups.

Type	pH	TA(°T)	WHC (%)	Syneresis (%)
L_1_	4.50 ± 0.01^a^	72.00 ± 2.65^b^	65.72 ± 2.83^a^	50.28 ± 0.20^b^
Kefir	4.52 ± 0.02^a^	77.67 ± 2.08^a^	44.13 ± 2.85^b^	60.23 ± 1.71^a^

Different letters in the same column indicate significant differences (*p* < 0.05).

The WHC of the kefir group was 32.85% lower than that of the L_1_ group. Firstly, yeast will produce ethanol by alcohol fermentation under anaerobic conditions due to the addition of yeast and acetic acid bacteria in the kefir group. The acetic acid bacteria will use ethanol to produce water. Water and whey are not easy to separate in yoghurt, which will also result in a lower water holding rate. In addition, the sensitivity of kefir to dehydration did not decrease because the fermentation time of the kefir sample was less than 18 h, which was 19.78% higher than that of the L_1_ group. The kefir treatment group showed low WHC and high levels of dehydration shrinkage, which were consistent with the results of Bensmira and Jiang (2012). Yoghurt prepared at low temperatures had a higher WHC than that at high temperatures, and the dehydration rate of the samples fermented at high temperatures was significantly increased.

### 3.3. Texture analysis

The texture of the kefir and L_1_ fermented milk was compared and analysed. The evaluation indexes mainly included hardness, elasticity, cohesion and so on. Table 2 shows that the hardness of the kefir group was 42.18% higher than that of the L_1_ group, and the viscosity was increased by 40.9%. This condition may be related to the electrostatic interaction between the protein matrix and TN_1_ and A_3_, forming an electrostatic complex, resulting in a dense network (Wang et al., 2022). The kefir group also significantly reduced the elasticity (30.93%) and cohesiveness (16.95%) of yoghurt (*p* < 0.05). This phenomenon may be caused by the addition of acetic acid bacteria after the yoghurt’s acidity increased, resulting in the disintegration of the gel structure. The addition of yeast resulted in reduced cohesion and viscosity, and decarboxylation has a certain effect on the texture and physical properties.

**Table 2:** Effects of L_1_ and kefir groups on the texture of yoghurt.

Type	L_1_	Kefir
Hardness	127.11 ± 6.65^b^	180.72 ± 3.26^a^
Elasticity	0.97 ± 0.008^a^	0.67 ± 0.06^b^
Cohesiveness	0.59 ± 0.016^a^	0.49 ± 0.048^b^
Stickiness	110.47 ± 3.21^b^	155.74 ± 4.20^a^
Resilience	0.03 ± 0.00^b^	0.09 ± 0.01^a^

Different letters in the same row indicate significant differences (*p* < 0.05).

In addition, the higher hardness and adhesion of the kefir group may be related to the fermentation temperature. Haque et al. (2001) pointed out that increasing the fermentation temperature would result in a systematic increase in the hardness, adhesion, and deformation resistance of the kefir milk. Second, the acetic acid bacteria will produce acetic acid acidification reaction (Bensmira and Jiang, 2012), which will result in cheese, and the hardness will be significantly improved. The hardness may also be related to the use of a high protein concentration of milk fermentation. Following a comprehensive evaluation, the addition of yeast and acetic acid bacteria will change the structure of yoghurt, in accordance with the characteristics of kefir yoghurt beverage.

### 3.4. Rheology analysis

**Figure 2:**
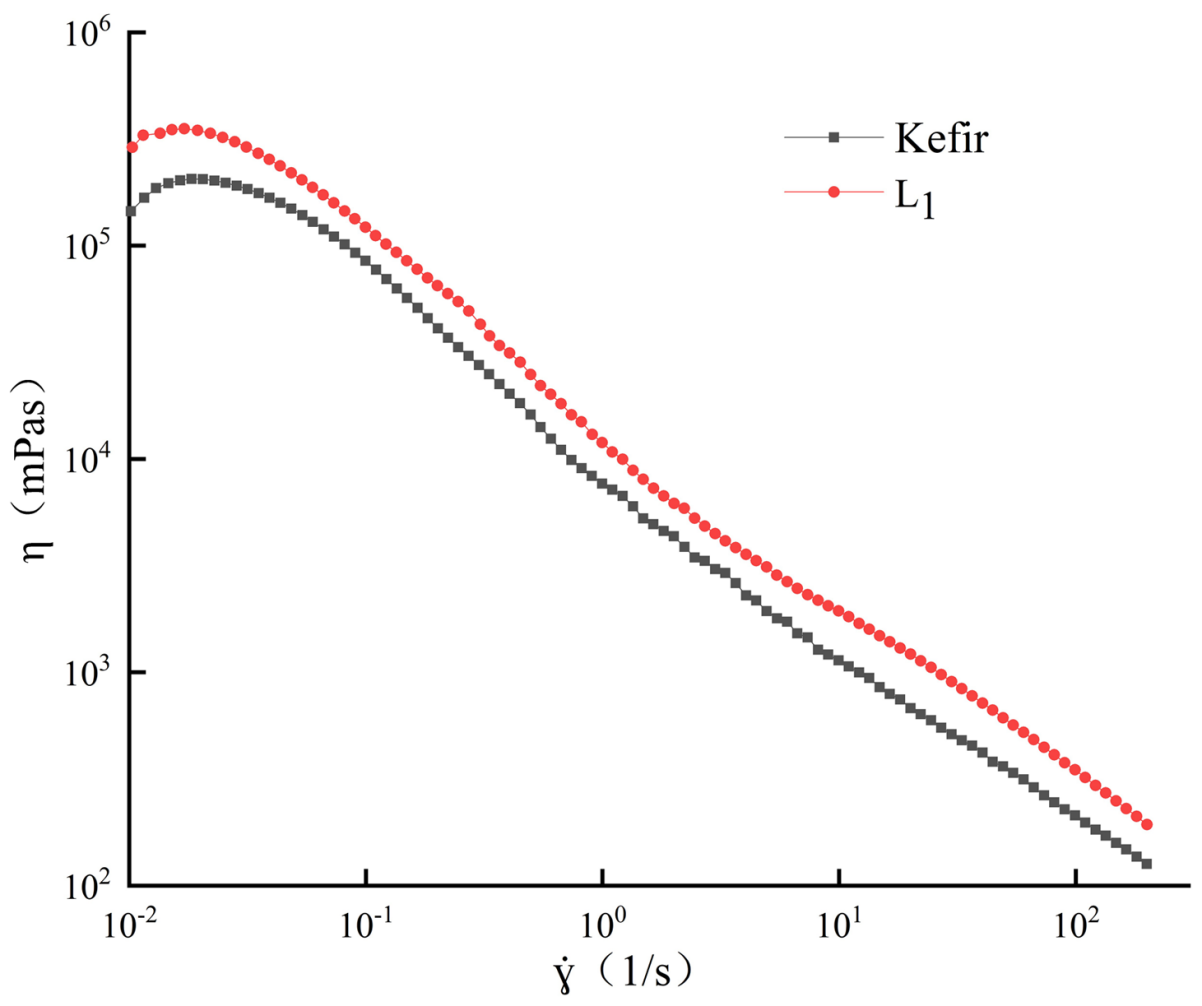
Analysis of rheological properties.

The apparent viscosity of the two groups of yoghurt in the shear range of 0.1–200 s^−1^ showed a shear dilution phenomenon with the increase in shear rate (Figure 2), and it gradually decreased. This shear thinning behaviour may be due to the destruction of intramolecular and intermolecular associations in the yoghurt system (Duboc and Mollet, 2001). The initial viscosity of the single strain L_1_ treatment group was slightly larger than that of the kefir treatment group. At a high shear rate, the apparent viscosity of the L_1_ fermented milk was always greater than that of kefir fermented milk, and the final viscosity was better. The L_1_ treatment group had a large number of lactic acid bacteria and strong metabolic activity, thereby promoting the binding of casein micelles by reducing the pH value, with better viscosity and less water loss in the matrix space.

No significant difference was observed between the curve of the kefir treatment group and the single strain L_1_ group. However, the greater viscosity consumption may be related to the increase in hardness during fermentation. This phenomenon may be due to the hydrolysis of casein leading to the plasticisation of water and the associated decrease in mechanical force viscosity (Alinovi et al., 2018), which weakens the resistance of yoghurt gels to breakdown. Meanwhile, this phenomenon is the result of the interaction of milk proteins adsorbed on the fat droplets. The degree of fat globule dispersion is large, and the number of free casein units that can form a network is reduced because the commercial milk after homogenisation is used. Moreover, the acidity of this group is high due to the addition of acetic acid bacteria. Thus, the protein structure is more easily destroyed.

### 3.5. Confocal laser scanning microscope (CLSM)

**Figure 3:**
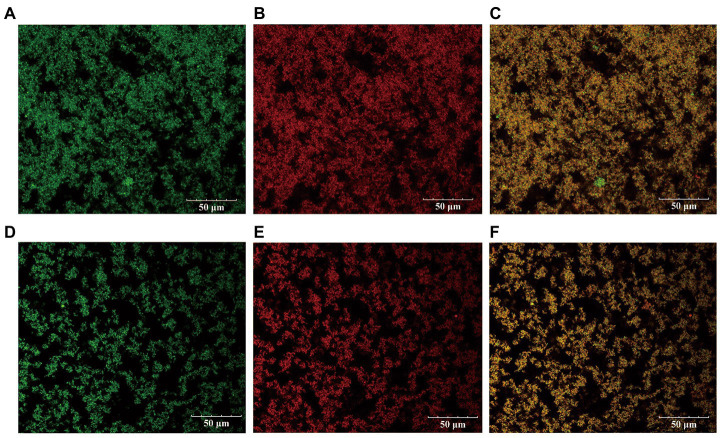
The distribution of protein and fat in L_1_ fermented milk and kefir fermented milk under laser scanning confocal. (**A** represents the fat distribution in L_1_, **B** represents the protein distribution in L_1_, and **C** is the superimposed effect of the two. **D** represents the fat distribution in kefir, **E** represents the protein distribution in kefir, and **F** is the superimposed effect of the two).

The microstructural difference between the kefir fermented milk and the L_1_ fermented milk was observed by CLSM (Figure 3). Fat (green) and casein clusters (red), yellow for the superposition of fat and protein renderings. Significant differences in the casein cluster structure can be observed between the L_1_ and the kefir groups. Both treatment groups showed a continuous flocculent protein structure. The flocculent protein structure of the kefir group was not as dense as that of the L_1_ group, the gap of the branch protein network was larger, the distribution was more dispersed, and the connection between groups was weaker. This condition may be related to the ability of the fat globules to positively interact with casein matrix and whey protein. Yoghurt gels with high whey protein ratio have a more discontinuous structure with larger pores, and the presence of large whey protein aggregates promotes the formation of coarse gel microstructures characterised by large gaps (Krzeminski et al., 2011). There are research reports that yeast and acetic acid bacteria co-ferment and decompose proteins in the substrate to produce vitamin B6, consume oxygen in the system to produce anaerobic environment, and produce acetic acid to reduce the pH of the system, thereby activating some lactic acid bacteria to ferment lactose and produce extracellular polysaccharides (Tao et al., 2022). In addition, The kefir group produces a cheese similar to the original kefir grains with increased acidity and more whey precipitation due to the synergistic action of multiple strains (Karim and Aider, 2022). The microstructure of low-fat yoghurt with a smaller gap of L_1_ protein cluster, higher proportion of whey protein and increased aggregation of whey protein was mainly a granular network. The gels with a high casein-to-whey ratio observed finer protein chains, smaller particle sizes, more uniform distribution, and stronger inter-group linkages (Figure 3B). This situation also explains the reason why the L_1_ group yoghurt has a higher WHC. Such a protein network structure is more conducive to increasing the WHC of the gel. The kefir group (Figure 3E) has a large gap, and the dispersed microstructure arrangement will lead to a weaker ability of the protein to intercept water molecules, low water holding capacity and increased dehydration capacity. This situation is also related to texture and rheology, and the gel is easier to loosen. The kefir group observed in SEM that casein is tightly cross-linked but does not have a stronger internal bond-stabilised complex, which may result in protein rearrangement during storage and an unstable casein network, which is also related to the rapid decrease in apparent viscosity in rheological properties.

### 3.6. SEM analysis of the yoghurt microstructure

**Figure 4:**
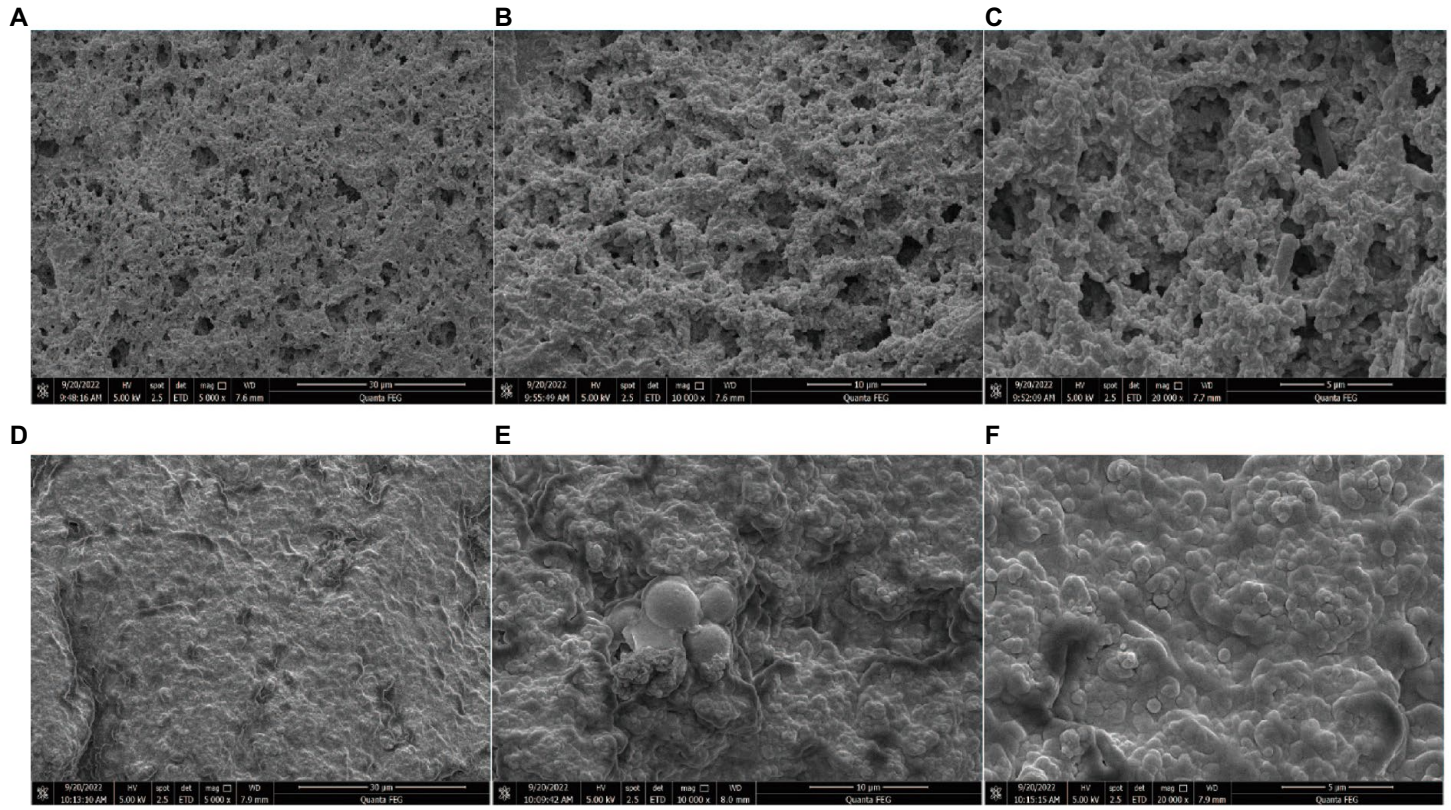
The SEM micrographs of yogurt L_1_ and kefir at different magnifications (**A,B,C** represents L_1_ yogurt magnification of 5.00 k×, 10.00 k×, and 20.00 k×. **D,E,F** represents kefir magnification of 5.00 k×, 10.00 k×, and 20.00 k×).

The microstructure of set yoghurt system was observed by scanning electron microscopy (SEM). The results are shown in Figure 4. The SEM revealed a smaller and more compact protein network in the two groups, which may be related to the use of milk with higher protein content before fermentation.

In the structure of the L_1_ group, the density of the gel network and molecular stacking increased, the pore size was continuous, the whey pores were evenly distributed, and the size was uniform. This condition is attributed to the aggregation of casein and denatured proteins on the micelle surface that results in the formation of clusters during the acidification of milk, thereby forming a 3D network (Ozcan-Yilsay et al., 2007). *L. plantarum* L_1_ appears on the protein surface between the fat and the protein layers or is suspended in yoghurt. The single-strain L_1_ group fermented under anaerobic fermentation conditions showed stronger WHC and finer structure, which is consistent with the study.

Evident differences can be observed in the microstructure between the kefir and the L_1_ groups. The surface of the kefir group was a dense membrane composed of irregularly interconnected chains of a casein micelle protein matrix structure. SEM shows an analogue of biofilm. A previous study showed that Lactobacillus and Acetobacter are important strains which produce kefir polysaccharides and form biofilms, while yeast plays a role in connecting and promoting this complex interphase network structure and environment. In addition, some bacteria that secrete biofilm polysaccharides, such as Lactobacillus, are attached to the surface of small molecule particles (Dong et al., 2018). The extracellular polysaccharides secreted by them can adhere to other microorganisms, bacteria and fungi can adhere and co-exist through direct interaction and interact with the components (proteins) in the fermentation substrate, eventually forming a relatively sealed space (Fontán et al., 2006). The physicochemical properties showed that kefir had higher acidity, resulting in thicker protein networks, smaller and irregular gaps and larger clumps (Figure 4F). On the one hand, the more balanced the binding of casein micelles to whey protein, the fewer network voids observed because of the higher the protein content. Inhomogeneous structures and large pores were observed in gels containing large protein aggregates and bound to whey protein-encapsulated casein micelles, which is consistent with the results observed by CLSM and surface observation (Vasbinder et al., 2004). On the other hand, the kefir-treated group appears to be more tightly packed, with smaller gaps and a protein network similar to that of cheese. Accordingly, less water is retained inside, and the dehydration rate increases (Ahmed and Bajwa, 2019). The chain in the figure denotes the aggregation and growth of individual particles, which may also be caused by the aggregation of adjacent chains. In addition, casein micelle fusion intolerant protein particles may also result in an obscured micelle profile. Bensmira and Jiang (2012) noted that kefir histones were more tightly fused together, and clusters became denser and thicker as the fermentation temperature increased above 32°C, forming a relatively concentrated structure (Figure 3D) and more tightly crosslinked structure and promoting dehydration. These properties affected the texture of kefir (Table 1). In addition, the ability of the protein in the kefir group to intercept water molecules became weaker, and the dehydration shrinkage rate became higher. The honeycomb holes can be evidently seen from the outside, which can also explain that the protein cross-linking is closer, but the dehydration is stronger. The high content of acidic ions changed the phenomenon of protein interaction, which had an important influence on the final structure and quality of yoghurt.

### 3.7. Lf-NMR

**Figure 5:**
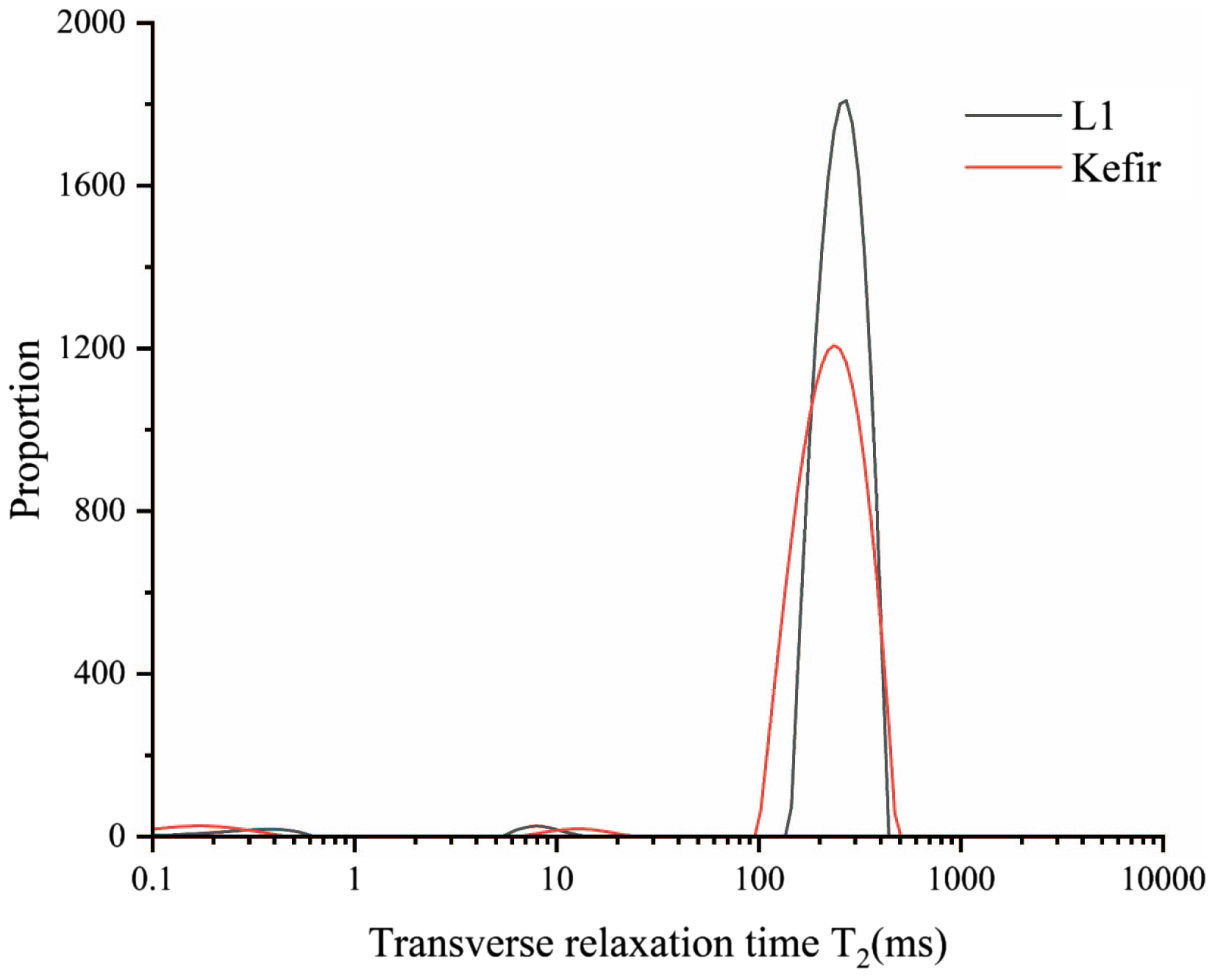
Distribution of T_2_ relaxation times on set yoghurts at L_1_ and kefir by LF-NMR.

The relaxation time T_2_ of yoghurt fermented by different probiotics was obtained based on the T_2_ relaxation time distribution map of the LF-NMR (Table 3). Relative signal intensities (T_21_, T_22_ and T_23_) represent bound water, semi-bound water and free water, respectively. The transverse relaxation time T_2_ of the two yoghurt treatment groups is shown in Figure 5. The LF-NMR transverse relaxation time T_2_ is divided into three regions: the T_21_ region with relaxation time between 0 ms and 3 ms represents the relaxation of hydrogen protons in the water molecule layer tightly bound to polar groups (i.e., bound water), which has the smallest fluidity; the T_22_ region with relaxation time between 5 ms and 30 ms represents immobile water. The T_23_ region with relaxation time between 100 ms and 500 ms represents free water (Liu et al., 2018; Xu et al., 2022).

**Table 3:** LF-NMR data of Kefir and L_1_.

Samples	Relaxation time(ms)			Peak area
	T_21_	T_22_	T_23_	T_23_
*L_1_*	0.633	9.479	304.661	18970.52
*Kefir*	0.478	20.996	409.786	17552.83

The results of relaxation time T_22_ showed that the relaxation time of the kefir group was longer than that of the L_1_ group. Table 3 illustrates that the kefir group had the smallest water distribution area and the lowest free water content in the T_23_ free water region, and the area of free water could reflect the water retention capacity of the protein gel structure (Xu et al., 2019). The kefir group may be due to the synergistic effect of yeast and acetic acid bacteria after the addition of yeast and acetic acid bacteria, which decomposes macromolecules, such as protein and fat, into small peptides and small lipid particles, fills the pores of the yoghurt gel structure, makes the pores smaller and forms a dense network structure that closely combines water molecules, similar to Wang et al. (2018). The free water in the kefir group retained less structure than the L_1_ group and was physically less capable of capturing water molecules. The LF NMR detection results are consistent with the experimental results of the microstructure, water retention and dehydration.

### 3.8. Amino acid analysis

**Figure 6:**
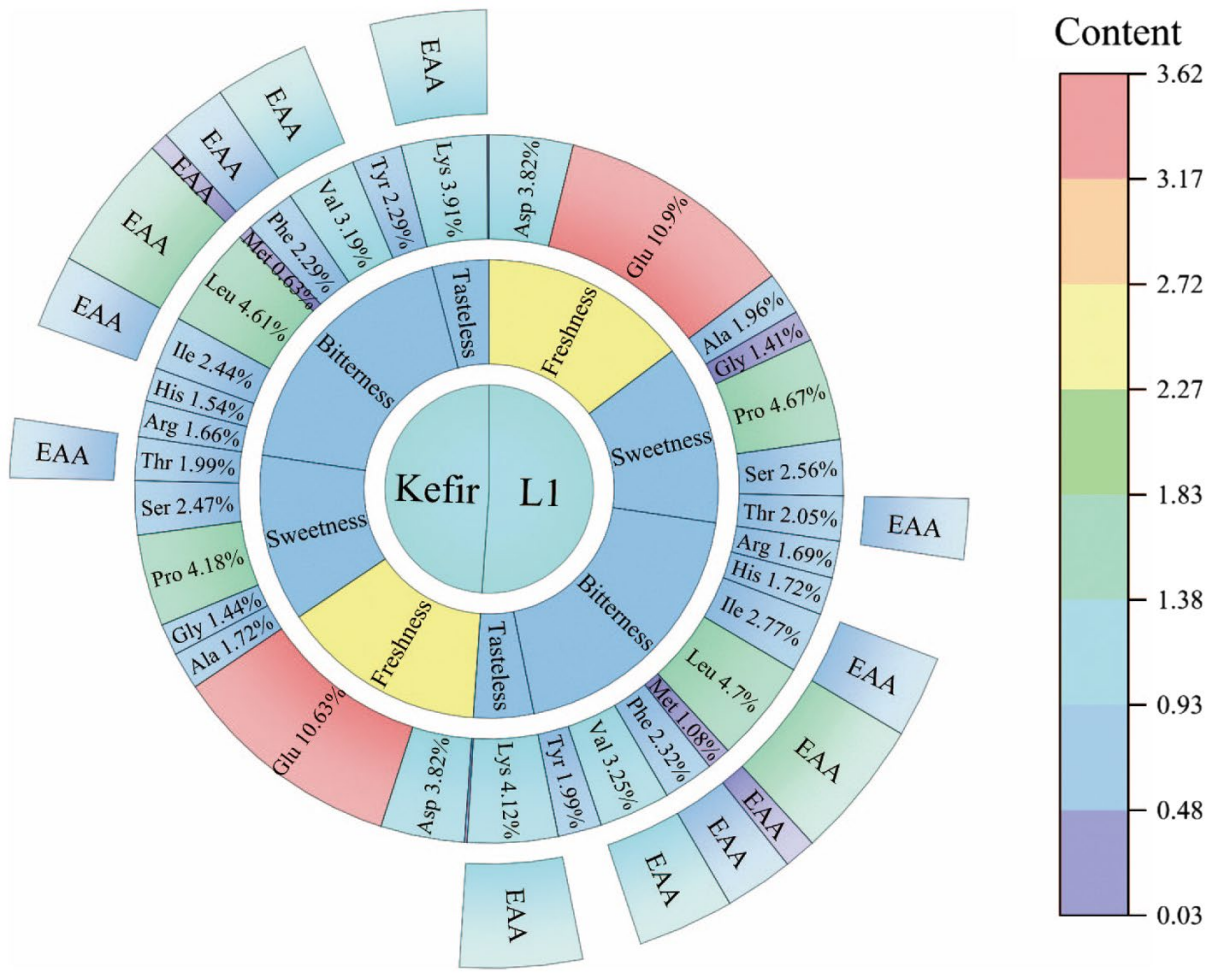
Sunburst diagram of amino acids of L_1_ and kefir.

Protein and amino acids are among of the important indicators of yoghurt. These indicators provide essential or non-essential amino acids and other nutrients to be utilised by the body (Day et al., 2021). Amino acids are also precursors of aromatic compounds in yoghurt, which give yoghurt different flavours (Celik et al., 2021). Yoghurt retains its freshness due to aspartic and glutamic acid. Threonine, serine, glycine, proline and alanine give yoghurt a pleasant sweetness. Leucine, isoleucine, phenylalanine and arginine exhibit bitterness (Cautela et al., 2021). In this study, 17 amino acids were detected in milk, yoghurt L_1_ and kefir. The results are shown in Figure 6. Glutamic acid is the most abundant amino acid in milk and yoghurt. A previous study reported that glutamic acid combines with ammonia in the body to form non-toxic glutamine, which reduces blood ammonia and the symptoms of hepatic coma (Jiang et al., 2022). Proline, aspartic acid, leucine, valine and lysine were the next most abundant. Amongst these amino acids, leucine and valine, which act as branched-chain amino acids, promote the release of insulin and growth hormone (Jung et al., 2021). Moreover, lysine, as an essential amino acid, can promote human growth and development and fat oxidation and enhance immunity, which has positive nutritional significance in many aspects (Ruocco et al., 2021).

The analysis found that the amino acid content of all yoghurts significantly changed compared with milk. Alanine, proline, threonine, isoleucine, methionine and lysine in kefir yoghurt were significantly lower than those in L_1_. The significant decrease in alanine, proline, isoleucine and methionine as hydrophobic amino acids was due to the addition of acetic acid bacteria and yeast, which may produce different flavour substances. However, whether this is the reason why the kefir yoghurt structure becomes a dense pore structure remains to be further studied. The amounts of glycine and tyrosine in kefir yoghurt increased by 1.02 and 1.15 times compared with those in L_1_. Glycine is a constituent amino acid of the endogenous oxidant glutathione (Rom et al., 2022). Liu et al. (2021) found that glycine improves the growth and proliferation of the neonatal pig intestinal epithelial cells and the ability to resist oxidative stress. In addition, the essential and non-essential amino acid content of the two yoghurts are shown in Table 4. Although the content of EAA in kefir is 0.94 times lower than that of L_1_, its EAA/NEAA exceeds 60%, and EAA/TAA reaches 39%, which is also a nutritional standard for high-quality protein.

**Table 4:** The contents of free acid in Kefir and L_1_ fermented yoghurt.

Classification	Content(mg/g)		FC	Change
	L_1_	Kefir		
EAA	6.74 ± 0.18^a^	6.34 ± 0.16^b^	0.94	–
NEAA	10.24 ± 0.21^a^	9.91 ± 0.19^b^	0.97	–
TAA	16.99 ± 0.39^a^	16.24 ± 0.35^b^	0.96	–
EAA/ NAA	65.82%^a^	63.98%^b^	0.97	–
EAA/TAA	39.70%^a^	39.01%^a^	0.98	–

The data are the means of three independent experiments ± standard deviations (*n* = 3). EAA is essential amino acid. NEAA is non-essential amino acid. TAA is total amino acid. FC represents the ratio of amino acid content of kefir and L1 in yoghurt. ^a,b^Values in the same row with different superscript letters differ significantly (*p* < 0.05).

### 3.9. Analysis of a flavour substance

A total of 33 flavour substances were detected by GC–MS, mainly esters, acids, ketones, aldehydes and alcohols. These substances include 12 esters, 6 ketones and aldehydes, 6 alcohols and 9 acids. Kefir had 6 more flavour substances than L1. The substance and relative content are shown in Table 5.

**Table 5:** Determination of volatile flavor compounds of L_1_ and kefir by GC–MS.

Classification	CAS	Formula	Comparative content(μg/mL)	
			L_1_	Kefir
Esters				
Ethyl caproate	C123660	C_8_H_16_O_2_	25.24 ± 0.41^b^	33.31 ± 1.31^a^
Diethyl succinate	C123251	C_8_H_14_O_4_	19.94 ± 0.55^a^	9.14 ± 0.19^b^
Phenylacetic acid ethyl ester	C101973	C_10_H_12_O_2_	3.57 ± 0.16^a^	0 ± 0.00 ^b^
Phenethyl acetate	C103457	C_10_H_12_O_2_	7.64 ± 0.20^a^	8.15 ± 0.29^a^
Butyldecalactone	C705862	C_10_H_18_O_2_	12.77 ± 0.24^b^	13.95 ± 0.58^a^
Ethyl palmitate	C628977	C_18_H_36_O_2_	133.63 ± 3.20^b^	194.84 ± 6.36^a^
Delta-Dodecalactone	C713951	C_12_H_22_O_2_	5.76 ± 0.20^a^	6.02 ± 0.09^a^
9-Octadecenoic acid, ethylest	C6114187	C_20_H_38_O_2_	10.99 ± 0.47^b^	15.19 ± 0.78^a^
Isoamyl acetate	C123922	C_7_H_14_O_2_	0 ± 0.00^b^	22.74 ± 1.23^a^
Lactic acid ethyl ester	C687478	C_5_H_10_O_3_	0 ± 0.00^b^	3.87 ± 0.18^a^
Ethyl ocanoate	C106321	C_10_H_20_O_2_	0 ± 0.00^b^	15.83 ± 0.66^a^
Ethyl salicylate	C118605	C_15_H_22_O_3_	0 ± 0.00^b^	3.09 ± 0.14^a^
Acids				
Acetic acid	C64197	C_2_H_4_O_2_	72.78 ± 3.32^b^	193.59 ± 20.15^a^
Butyric acid	C107926	C_4_H_8_O_2_	16.22 ± 0.60^a^	15.92 ± 0.64^a^
Isovaleric acid	C503742	C_5_H_10_O_2_	0 ± 0.00^b^	17.16 ± 0.62^a^
Octanoic acid	C124072	C_8_H_16_O_2_	218.53 ± 2.81^b^	353.56 ± 18.82^a^
Decanoic acid	C334485	C_10_H_20_O_2_	105.10 ± 2.29^b^	132.29 ± 1.77^a^
Lauric acid	C143077	C_12_H_24_O_2_	12.15 ± 0.37^b^	14.62 ± 0.52^a^
Palmitic acid	C57103	C_16_H_32_O_2_	4.66 ± 0.21^a^	5.07 ± 0.22^a^
Hexanoic acid	C142621	C_6_H_12_O_2_	138.29 ± 5.31^b^	155.51 ± 6.46^a^
Benzoic acid	C65850	C_7_H_6_O_2_	0 ± 0.00^b^	3.06 ± 0.09^a^
Alcohols				
2-Octanol	C5978701	C_8_H_18_O	54.38 ± 1.43^a^	52.03 ± 2.46^a^
1-Undecanol	C112425	C_11_H_24_O	10 ± 0.00^a^	9.23 ± 0.31^b^
Benzyl alcohol	C100516	C_7_H_8_O	14.33 ± 0.58^a^	11 ± 0.00^b^
Phenylethyl alcohol	C60128	C_8_H_10_O	7.64 ± 0.30^b^	115.74 ± 5.62^a^
Isobutyl alcohol	C763326	C_4_H_10_O	0 ± 0.00^b^	15.00 ± 0.63^a^
3-Methyl-1-butanol	C123513	C_5_H_12_O	0 ± 0.00^b^	237.98 ± 10.46^a^
Aldoketones				
Hexanal	C66251	C_6_H_12_O	3.78 ± 0.16^b^	4.71 ± 0.25^a^
2-Heptanone	C110430	C_7_H_14_O	46.20 ± 0.34^a^	28.32 ± 0.66^b^
Acetoin	C513860	C_4_H_8_O_2_	6.52 ± 0.26^b^	53.39 ± 1.14^a^
2-Nonanone	C821556	C_9_H_18_O	15.22 ± 0.52^a^	10.83 ± 0.54^b^
1-Nonanal	C124196	C_9_H_18_O	13.50 ± 0.11^b^	14.28 ± 0.41^a^
Decylaldehyde	C112312	C_10_H_20_O	6.64 ± 0.28^a^	6.78 ± 0.36^a^

The data are the means of three independent experiments ± standard deviations (*n* = 3). ^a,b^Values in the same row with different superscript letters differ significantly (*p* < 0.05).

**Figure 7:**
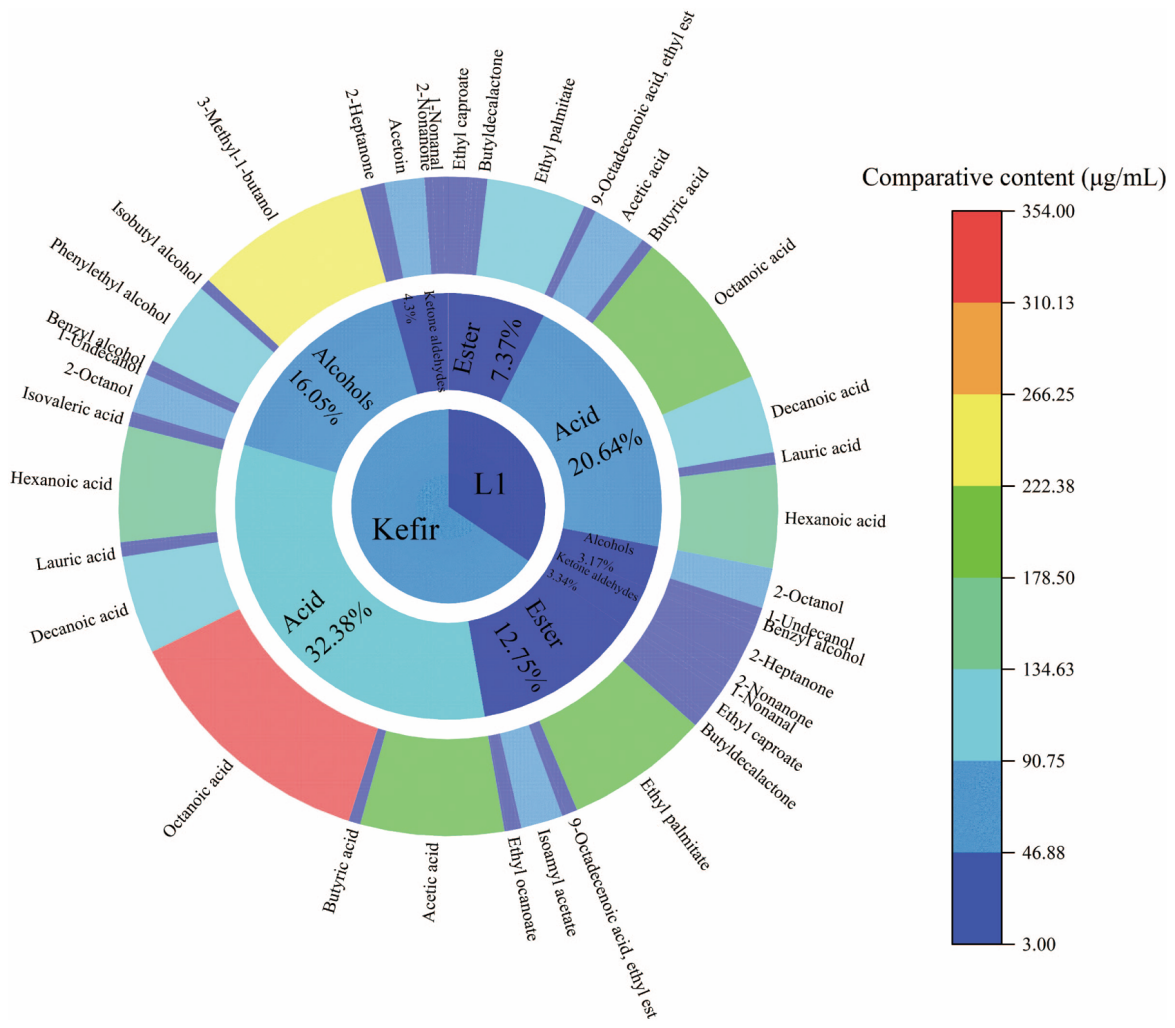
Sunburst diagram of flavor substances of L_1_ and kefir.

Flavour substances are produced by proteins, lactose and lipids through a series of metabolic pathways such as lipid decomposition, proteolysis and glycolysis (McAuliffe et al., 2019). The protein and fat in dairy products are metabolised by the enzyme system of the bacteria to produce flavour substances through the mixed culture of lactic acid bacteria, yeast and acetic acid bacteria, which affect the amino acid catabolism. Lactic acid bacteria decompose the biological macromolecules into small product molecules and add a certain special functional flavour to the final. Lactic acid bacteria decompose lactose into monosaccharides, produce lactic acid and provide carbon sources for the growth of yeast and acetic acid bacteria, promoting alcohol fermentation to produce special flavour substances, such as ethanol, CO_2_, organic acids and aldehydes (De Vuyst and Leroy, 2020). The flavor profile and content of L_1_ and kefir are shown in Figure 7. Kefir mainly detected octyl salicylate, isobutanol, ethyl lactate, isoamyl acetate, isovaleric acid, benzoic acid and ethyl caprylate, whilst the L_1_ group did not produce special substances. Ethyl caprylate and isovaleric acid are the unique flavours produced by kefir to increase the fruity flavour of yoghurt. Lauric and palmitic acids are saturated higher fatty acids to protect the liver. Saturated fatty acids can not only protect the liver from alcohol damage and give energy to the body (fatty acid synthesis is necessary to supplement the body’s energy consumption) but also increase the activity of some enzymes in the body. Ethyl lactate is a new product-ethyl lactate from lactic acid and ethanol. Acetaldehyde is converted into acetic acid and ethanol to produce malt aroma substance-isobutanol with the help of yeast, giving kefir unique soft mellow aroma. Furthermore, the higher content of two higher alcohols, phenylethyl alcohol and isoamyl alcohol in kefir, gave the typical rose aroma and fruit aroma in kefir fermented dairy products, respectively. Isobutanol, phenylethyl alcohol and isoamyl alcohol were rarely detected in ordinary yoghurt. The contents of 2-nonanone, 2-heptanone, nonanal, caprylic acid and acetoin significantly increased, which increased the cream flavour, proving that kefir was more palatable and had a special flavour that traditional kefir fermented yoghurt did not have. Therefore, the kefir treatment group can produce isobutanol, phenylethanol and isoamyl alcohol, which is of great significance to improve the flavour quality of kefir.

## 4. Conclusion and future research direction

This study found that L_1_ and kefir fermented milk were pseudoplastic fluids, but their rheological properties and microstructure were affected to varying degrees after the addition of yeast and acetic acid bacteria. Meanwhile, the acidity increased following the addition of *A. tropicalis* A_3_ and *S. cerevisiae* TN_1_. Consequently, the protein structure denatured, and the isoelectric point decreased. During the high-temperature fermentation, kefir histone cross-linking becomes stronger, and the forming part of cheese, fat and protein binding is non-uniform. The apparent viscosity and viscoelasticity are significantly reduced with the increase in shear force, and the structure is not sufficiently stable. The fermented milk beverage is prepared by evenly stirring, which is conducive to removing the non-smooth feeling of cheese particles, resulting in a taste close to champagne and a smooth entrance. L_1_ is more suitable for the preparation of set yoghurt. The gap between the L_1_ protein clusters is smaller, the distribution is more uniform, and the gel water holding capacity is better. The results of the amino acid analysis showed no significant difference between the kefir and the L_1_ groups, and the nutritional value was not affected. The decrease of some amino acid content was related to the flavour substances produced later. Compared to the L_1_ group, the kefir group was supplemented with isobutanol, phenylethyl alcohol and isoamyl alcohol, consistent with the special flavors detected in traditionally made kefir milk (giving rose, fruit and malt flavors, mellow and champagne flavors).

The change of microstructure has a great influence on the texture of kefir yoghurt, but it is not enough to analyse the microstructure to determine the quality of kefir yoghurt. We will study the metabolites, use metabolomics to summarise and analyse the characteristic metabolites and find the law of the difference between the two. Furthermore, we explore whether the characteristic metabolites have a certain influence on macromolecules, such as proteins and polysaccharides. Kefir’s industrial production, quality control and transportation storage has a certain degree of influence for the market to produce a real champagne yoghurt.

## Data availability statement

The original contributions presented in the study are included in the article/supplementary material, further inquiries can be directed to the corresponding author.

## Author contributions

RX: data curation, conceptualization, writing – review and editing, formal analysis, and supervision. ML: writing – review and editing, visualization, and supervision. XS, QT, and XH: conceptualization, and writing – review and editing. MH: writing – review and editing, funding acquisition, project administration, and supervision. All authors contributed to the article and approved the submitted version.

## Funding

This work was financially supported by the research and application of microflora of yujiu, a sub-project of major science and technology projects of Henan province, China (181100211400–8) and Henan University of Technology (No. 31401184).

## Conflict of interest

The authors declare that the research was conducted in the absence of any commercial or financial relationships that could be construed as a potential conflict of interest.

## Publisher’s note

All claims expressed in this article are solely those of the authors and do not necessarily represent those of their affiliated organizations, or those of the publisher, the editors and the reviewers. Any product that may be evaluated in this article, or claim that may be made by its manufacturer, is not guaranteed or endorsed by the publisher.
